# Comparing Thickness and Doping-Induced Effects on the Normal States of Infinite-Layer Electron-Doped Cuprates: Is There Anything to Learn?

**DOI:** 10.3390/nano12071092

**Published:** 2022-03-26

**Authors:** Chiara Sacco, Alice Galdi, Francesco Romeo, Nunzia Coppola, Pasquale Orgiani, Haofei I. Wei, Kyle M. Shen, Darrell G. Schlom, Luigi Maritato

**Affiliations:** 1 DIIN, Università degli Studi di Salerno, 84084 Fisciano, Italy; chsacco@unisa.it (C.S.); lmaritato@unisa.it (L.M.); 2 CNR SPIN, UOS Salerno, 84084 Fisciano, Italy; 3 CLASSE, Cornell University, Ithaca, NY 14583, USA; agaldi@unisa.it; 4 Dipartimento di Fisica “E.R. Caianiello”, Università degli Studi di Salerno, 84084 Fisciano, Italy; fromeo@unisa.it; 5 TASC Laboratory, Istituto Officina dei Materiali (IOM)-CNR, Area Science Park, S.S. 14, Km 163.5, 34149 Trieste, Italy; orgiani@iom.cnr.it; 6 Department of Physics, Cornell University, Ithaca, NY 14853, USA; hw437@cornell.edu (H.I.W.); kmshen@cornell.edu (K.M.S.); 7 Kavli Institute at Cornell for Nanoscale Science, Ithaca, NY 14853, USA; schlom@cornell.edu; 8 Department of Material Science and Engineering, Cornell University, Ithaca, NY 14853, USA

**Keywords:** infinite layer, electron-doped cuprates, normal-state properties

## Abstract

We grew Sr_1-x_La_x_CuO_2_ thin films and SrCuO_2_/Sr_0.9_La_0.1_CuO_2_/SrCuO_2_ trilayers by reflection high-energy diffraction-calibrated layer-by-layer molecular beam epitaxy, to study their electrical transport properties as a function of the doping and thickness of the central Sr_0.9_La_0.1_CuO_2_ layer. For the trilayer samples, as already observed in underdoped SLCO films, the electrical resistivity versus temperature curves as a function of the central layer thickness show, for thicknesses thinner than 20 unit cells, sudden upturns in the low temperature range with the possibility for identifying, in the normal state, the T* and a T** temperatures, respectively, separating high-temperature linear behavior and low-temperature quadratic dependence. By plotting the T* and T** values as a function of T_C_^onset^ for both the thin films and the trilayers, the data fall on the same curves. This result suggests that, for the investigated trilayers, the superconducting critical temperature is the important parameter able to describe the normal state properties and that, in the limit of very thin central layers, such properties are mainly influenced by the modification of the energy band structure and not by interface-related disorder.

## 1. Introduction

Advances in oxide thin film deposition processes and, in particular, in oxide molecular beam epitaxy (O-MBE) techniques [1,2,3,4,5] allowing atomic-scale thickness control, abrupt interfaces, and the possibility to change the chemical composition over a distance of a single-unit cell, have given a fundamental burst to the study of the low-dimensional effects in oxide-based heterostructures [6,7,8,9], allowing the fabrication of these systems with performances comparable to those of the best conventional semi-conductor devices. In particular, several oxide-based quantum-well (QW) systems, where a thin conducting oxide is sandwiched between two layers of insulating oxide material, such as SrTiO_3_/SrVO_3_/SrTiO_3_, GdTiO_3_/SrTiO_3_/GdTiO_3_, SrVO_3_/SrTiO_3_/SrVO_3_, and SmTiO_3_/SrTiO_3_/SmTiO_3_ [10,11,12,13,14], have been fabricated and studied. In the case of oxide-based QWs, it is highly desirable to have the possibility of varying both the thickness of the central layer and its charge carrier density. Moreover, it is convenient, especially from an experimental perspective, to minimize the number of different materials (sources) involved in the deposition. Finally, as many TMOs are Mott insulators with small energy band gaps, the question arises whether they can effectively be used to obtain charge confinement. Recently, results obtained on p-type SrMnO_3_/La_0.7_Sr_0.3_MnO_3_/SrMnO_3_ trilayers confirm charge confinement, pointing out that a small gap Mott insulator, such as SrMnO3 (Eg = 0.35 eV) [15,16], can be successfully used in QW structures as a barrier. However, a critical point in the obtainment of affordable oxide-based QWs is related to the control of their transport properties intervening directly on parameters such as the charge carrier density and the energy band structure without being limited by interface disorder effects.

In the view of the fabrication of affordable oxide-based QWs, we grew Sr_1−x_La_x_CuO_2_ (SLCO) thin films and (SrCuO_2_)/(Sr_0.9_La_0.1_CuO_2_)/(SrCuO_2_) trilayers in order to study and compare their electrical transport properties as a function of both the n-doping and the thickness of the central Sr_0.9_La_0.1_CuO_2_ layer. SrCuO_2_ has an energy gap higher than 1.2 eV [17] and, therefore, for similar interface roughness and band alignment, should provide even better confinement of the charge carriers when compared with SrMnO_3_ with a similar thickness. Moreover, the elemental sources needed to obtain the (SrCuO_2_)/(Sr_0.9_La_0.1_CuO_2_)/(SrCuO_2_) trilayers are only three and, by playing with the stoichiometric ratio between Sr and Mn, it is, in principle, possible to easily change the central layer charge carrier density.

By comparing the electrical resistivity versus temperature curves of the SLCO thin films as a function of the doping level (i.e., the La concentration x) to those of the (SrCuO_2_)/(Sr_0.9_La_0.1_CuO_2_)/(SrCuO_2_) trilayers as a function of the Sr_0.9_La_0.1_CuO_2_ central layer thickness, we were able to find many strong similarities. In particular, in the normal state of both types of samples, at temperatures higher than T*, a doping/thickness dependent temperature value, linear behaviors of the electrical resistivity versus temperature curves were observed. Meanwhile, at temperatures lower than T**, dependent on the samples’ doping/thickness, a quadratic dependence was obtained. We discuss the observed results, in terms of the common effects caused by the doping and central layer thickness, evidencing that, in the case of very thin films of SLCO, the transport properties are mainly influenced by intrinsic parameters (charge carrier density, energy band structure) and not by interface disorder.

## 2. Materials and Methods

Both the SLCO thin films and the (SrCuO_2_)/(Sr_0.9_La_0.1_CuO_2_)/(SrCuO_2_) trilayers were grown in a Veeco-GEN10 dual-chamber oxide MBE system using a shuttered layer-by-layer deposition process performed in purified O_3_ at a background pressure of 3 × 10^−7^ Torr. The films were deposited on (1 1 0) TbScO_3_ (TSO) substrates, which have an orthorhombical distorted perovskite structure (pseudo-cubic lattice parameter 0.3958 nm [18]). Both the SLCO and the SCO layers (with bulk in-plane lattice parameters 0.3951 nm [19] and 0.3927 nm [20], respectively) grown on TSO were subject to tensile strain. Details on the adopted deposition procedure can be found in [21]. Here, we identify that the after-growth in situ annealing step and the substrate-induced tensile strain are crucial for obtaining the infinite-layer (IL) phase without the presence of apical oxygen in the crystal structure [22,23,24,25].

The crystalline quality of the SLCO films as well as the in-plane and the out-of-plane lattice parameters were evaluated by HL-planes reciprocal space maps (RSMs) around the symmetrical (002) and asymmetrical (103) Bragg reflections and X-ray reflectivity (XRR) analysis were performed to measure the final film thickness. The structural quality of the obtained Sr_1−x_La_x_CuO_2_ films was also analyzed by scanning transmission electron microscopy (STEM) [26]. XRR and XRD analysis were also used to structurally characterize the (SrCuO_2_)/(Sr_0.9_La_0.1_CuO_2_)/(SrCuO_2_) trilayered samples. The XRR measurements give information about the thickness of the central layers in the heterostructure, and about the interface roughness, and confirm the validity of the adopted thickness calibration procedure, showing bottom SCO thickness to be 20 unit cell (u.c.) and the top SCO layer to be 15 u.c. thick, while the SLCO thickness varied from *t* = 20 to *t* = 5 u.c. [21].

All the electrical transport property measurements were performed on un-patterned samples using a van der Pauw four-probe geometry [27]. To make ohmic contacts, the samples were scratched on the four corners, and the four points scratched were covered with pressed indium. The resistivity of the samples was estimated as:

(1) $$\rho \left(T\right)=R_{Sh}\cdot t_{SLCO}$$

where *R_Sh_* is the sheet resistance value obtained by the van der Pauw geometry, and *t_SLCO_* is the thickness of the SLCO films or of the SLCO central layer.

## 3. Results and Discussion

**Figure 1:**
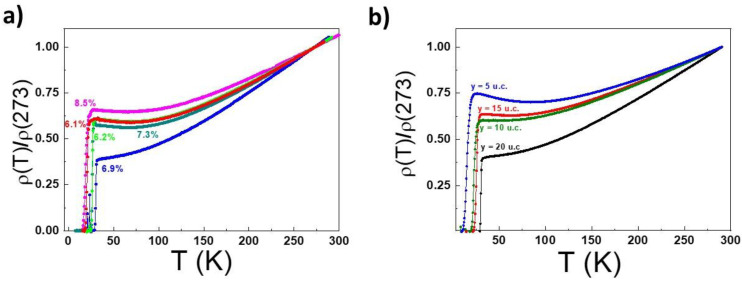
(**a**) Room temperature normalized resistivity vs. temperature ρ(T) curves for SLCO films at different doping levels; (**b**) room temperature normalized resistivity vs. temperature ρ(T) curves for trilayers with different central layer thickness.

In Figure 1a,b, the temperature dependence of the room temperature normalized electrical resistivity *ρ* is presented for the SLCO films and the (SrCuO_2_)/(Sr_0.9_La_0.1_CuO_2_)/(SrCuO_2_) trilayered samples, respectively. The room temperature values of the resistivity of all the investigated samples were in the range 0.1–0.5 mΩ cm. It is clear from Figure 1a,b that the very similar behaviors shown by the SLCO *ρ* (T) curves are a function of the doping, and those of the trilayers are a function of the thickness of the central layers. In both the figures, at high temperatures, the curves show a linear behavior with no sign of saturation. By defining T* as the lowest temperature at which the *ρ* (T) linear behavior is observed, T* values dependent upon the doping and the central layer thickness are obtained, see Table 1. At low temperatures, the curves in both Figure 1a,b show an upturn in the *ρ* values, which become more evident by decreasing the doping or the central layer thicknesses. If we define T_min_ as the temperature at which the local minimum in the *ρ*(T) curves is located, for all the investigated samples, the T_min_ values fall in the temperature range between 49 and 83 K (Table 1). The superconducting critical temperature T_c_^onset^, defined as the temperature at which *ρ* is 90% of its value just before the superconducting transition starts (the local maximum at low temperatures), shows a clear decreasing dependence as a function of decreasing doping values or central layer thicknesses. Recently [28], in the case of SLCO thin films with low levels of doping, a hidden Fermi-liquid (FL) charge transport was highlighted, in agreement with other similar things both in n-doped [28] and in p-doped [29,30] superconducting cuprates. In particular, assuming the low temperatures upturn to be phenomenologically associated to a logarithmic term, the SLCO *ρ*(T) curves were described using the formula

(2) $$\rho \left(T\right)=A_{0□}-A_{log□}\cdot \log(1/1K)+A_{2□}T^{2}\;$$

The good agreement with the experimental data, up to doping dependent temperatures T**, has been interpreted as a first indication of the possible presence of FL charge transport in these compounds. Such an FL regime has also been confirmed by Hall measurements, showing the cotangent of the Hall angle, cot(θ_H_), to follow a T^2^ law independent to doping with no appreciable changes up to T** [28]. For both the hole and the electron-doped cuprates, the coefficient $A_{2□}$ shows similar values at the same level of doping and is inversely proportional to the charge carrier concentration [27]. The data of T_c_^onset^, T*, and T** in Table 1, relative to the SLCO films, are summarized in Figure 3a. The red and black dashed lines in Figure 3a are guides to the eyes. A similar correlation between these quantities was found for almost all the superconducting cuprate compounds, although their physical interpretation is still controversial. As a very general trend, it can be said that all the different theoretical explanations are mainly based on the redistribution effects of the charge carriers’ density on the energy band structure, and not on extrinsic mechanisms, such as disorder or others [31,32,33,34].

**Figure 2:**
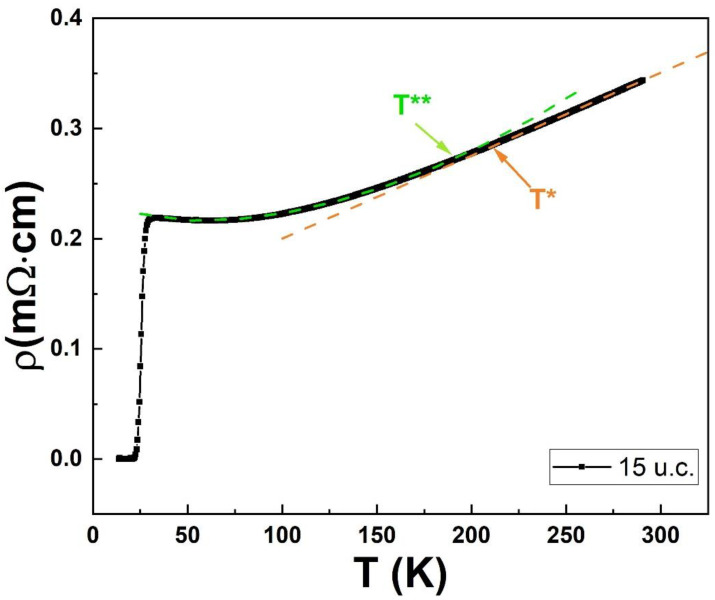
*ρ*(T) curve for the trilayer with the 15 u.c. thick central layer. The meaning of the T* and T** temperatures is discussed in the text.

As already indicated, the strong similarities in Figure 1a,b suggest using the same approach seen in the case of SLCO films to also analyze the *ρ*(T) curves obtained for the (SrCuO_2_)/(Sr_0.9_La_0.1_CuO_2_)/(SrCuO_2_) trilayers. In Figure 2, to better show the way our analysis has been performed, we show the *ρ*(T) curve for the trilayer with the 15 u.c. thick central layer. The orange line is the best linear fit obtained in the high temperature range and the temperature T*, indicated by an orange arrow, is the temperature at which the fit starts to deviate from the experimental data (using a 1% criterion).

**Figure 3:**
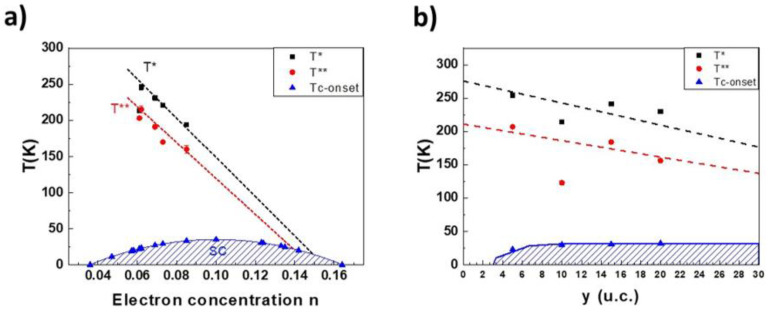
(**a**) T_C_^onset^, T*, and T** data in Table 1 for SLCO films as a function of the doping; (**b**) T_C_^onset^, T* and T** data in Table 1 for trilayers as a function of the thickness of the central layer. The red and black dashed lines are guides to the eyes.

The green line in Figure 2 is the best fit to the low temperature data obtained by Equation (2) and T**, indicated by the green arrow which is the temperature where, as in the T* case, the deviation from the experimental points starts to be larger than 1%. The data of T_c_^onset^, T*, and T**, relative to the investigated trilayered samples reported in Table 1, are summarized in Figure 3b.

Comparing Figure 3a,b, one can notice that effects due to the thickness of the central layer seem to play a role very similar to those induced by the doping. Moreover, the $A_{2□}$ values obtained by fitting Equation (2) with the low temperatures *ρ*(T) curves of the (SrCuO_2_)/(Sr_0.9_La_0.1_CuO_2_)/(SrCuO_2_) trilayers, are very close to those obtained in the case of SLCO films at different doping levels, see Table 1, and seem to have a trend of decreasing with the increasing thickness of the central layer.

When confining charge carriers in a trilayer system, as in the one presently investigated, the question arises if such a confinement is due to disorder effects introduced by the interfaces or to the modification of the charge carrier density distribution in the energy band structure approaching a two-dimensional limit. Recently [20], for (SrCuO_2_)/(Sr_0.9_La_0.1_CuO_2_)/(SrCuO_2_) trilayers, it has been shown that the observed dependence of T_C_^onset^ versus the thickness of the central layer, generally interpreted by using a model valid for superconducting thin films with order parameter leakage at the interface [35], is better described in terms of a Ginzburg–Landau approach with conventional (hard-wall) boundary conditions, appropriate to the case of a system not perturbed by interface effects. Moreover, the effects of the doping level on the pseudo-gap region boundary and on the superconducting transition in LSCO thin films have been deeply investigated using angle-resolved photoemission spectroscopy measurements [36]. The similarity between the effects on the transport properties of the doping and of the central layer thickness seems to confirm, in the trilayer samples investigated in this work, the prevalent presence of charge carrier density modification induced by approaching a two-dimensional limit respect to interface-related disorder effects.

**Figure 4:**
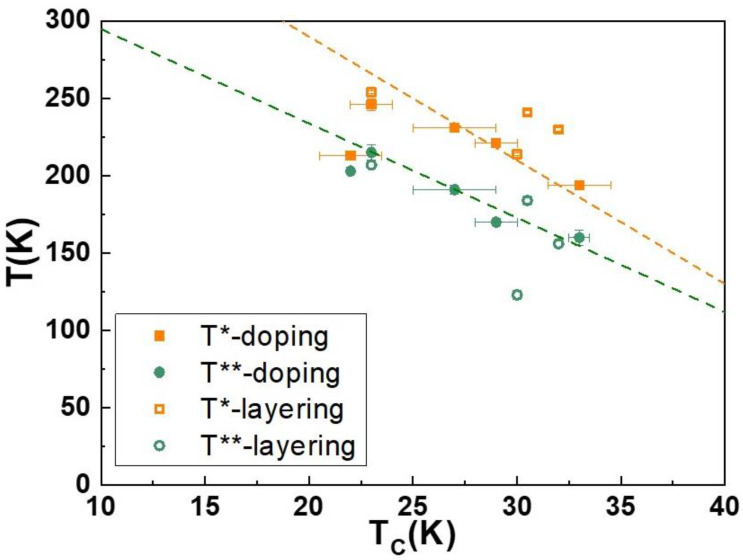
T* and T** values for the SLCO films and trilayers as a function of T_Conset_. The dotted lines are guides for the eyes.

To summarize, the observed behaviors of both the Sr_1−x_La_x_CuO_2_ films, as a function of doping, and of the SrCuO_2_/Sr_0.9_La_0.1_CuO_2_/SrCuO_2_ trilayers, as a function of the central layer thickness, in Figure 4 we have plotted the T* and T** data for the films and the trilayers as a function of the onset superconducting critical temperatures T_C_^onset^. Surprisingly, in this plot, the T* and T** curves, followed by the layering and the doping dependences, are the same. This observation clearly indicates that the superconducting critical temperature T_c_ is the unique and important parameter able to describe the effects due to the central layer thickness and the doping on the normal-state electrical transport properties of the investigated systems. Moreover, in superconducting systems, changes in T_c_ are generally ascribed to disorder or to a variation of the change carriers’ density. In Sr_1−x_La_x_CuO_2_ films, the observed dependence of T_c_ with doping is typically traced back to a change in the carrier density values at the Fermi surface, with disorder playing only a minor role. The fact that the SrCuO_2_/Sr_0.9_La_0.1_CuO_2_/SrCuO_2_ trilayers as a function of the central layer thickness are also following the same T_c_ dependence of T* and T** observed for Sr_1−x_La_x_CuO_2_ films as a function of doping, strongly suggests that the main effect induced by the decrease in the central layer thickness is not related to interface disorder effects but to the decreased influence on the charge carriers’ density distribution along the energy band structure in the limit of the very thin (almost two-dimensional) central layer.

## 4. Conclusions

Using an oxide-MBE deposition technique we have grown Sr_1−x_La_x_CuO_2_ epitaxial thin films with different doping levels and SrCuO_2_/Sr_0.9_La_0.1_CuO_2_/SrCuO_2_ trilayers with different thicknesses of the central layer. The normal-state electrical transport properties of both these systems present a high-temperature resistivity linear dependence and a low-temperature quadratic behavior characterized by temperatures T* and T** (T* > T**), respectively, which are functions of the doping in the thin films and of the thickness in the trilayers. Doping and the central layer thickness also influence the superconducting critical temperature T_C_^onset^. Our results show that, by plotting the T* and T** values as a function of T_C_^onset^ for both the thin films and the trilayers, the data fall on the same curves, clearly indicating, at least for the investigated Sr_1−x_La_x_CuO_2_ epitaxial thin films and SrCuO_2_/Sr_0.9_La_0.1_CuO_2_/SrCuO_2_ trilayers, that the superconducting critical temperature T_C_ is the single and important parameter to describe the normal-state properties of the investigated samples. Although, further systematic studies are still needed, the obtained results suggest that the decrease in the central layer thickness plays a role in the transport properties by influencing the charge carrier distribution in its energy band structure, and not by introducing interface-related disorder in the system.

## Figures and Tables

**Table 1:** **:** T_min_, T_C_^onset^, T*, T** and $A_{2□}$ values obtained from resistivity measurements for each of the examined samples.

	T_min_ (K)	T_C_^onset^ (K)	T* (K)	T** (K)	$A_{2□}(m\Omega \cdot K^{-2})$
Doping level					
6.1%	69	22	213 ± 3	203 ± 1	28.74 ± 0.03
6.2%	64	23	246 ± 4	215 ± 5	40.35 ± 0.03
6.9%	69	27	231 ± 2	191 ± 3	33.35 ± 0.003
7.3%	65.5	29	221 ± 1	170 ± 1	34.268 ± 0.009
8.5%	-	33	194 ± 2	160 ± 1	36.47 ± 0.06
SLCO Thickness					
5 u.c.	81	23	254 ± 1	207 ± 2	47.9 ± 0.1
10 u.c.	64	30	214 ± 2	123 ± 3	103.2 ± 0.9
15 u.c.	48	30.5	241 ± 1	184 ± 0.5	67.4 ± 0.2
20 u.c.	45	32	230 ± 1	156 ± 2	32.8 ± 0.06
